# Catalytic oxidation of CO over mesoporous copper-doped ceria catalysts *via* a facile CTAB-assisted synthesis

**DOI:** 10.1039/c8ra02327a

**Published:** 2018-04-19

**Authors:** Hongjian Zhu, Yingying Chen, Zhongpeng Wang, Wei Liu, Liguo Wang

**Affiliations:** School of Resources and Environment, Key Laboratory of Water Resources and Environmental Engineering in Universities of Shandong, University of Jinan 336 Nanxinzhuangxi Road Jinan 250022 PR China chm_wangzp@ujn.edu.cn stu_liuw@ujn.edu.cn

## Abstract

Nanosized copper-doped ceria CuCe catalysts with a large surface area and well-developed mesoporosity were synthesized by a surfactant-assisted co-precipitation method. The prepared catalysts with different Cu doping concentrations were characterized by XRD, DLS analysis, TEM, BET, Raman, H_2_-TPR and *in situ* DRIFTS techniques. The influence of Cu content on their catalytic performance for CO oxidation was also studied. The XRD results indicate that at a lower content, the Cu partially incorporates into the CeO_2_ lattice to form a CuCe solid solution, whereas a higher Cu doping causes the formation of bulk CuO. Copper doping favors an increase in the surface area of the CuCe catalysts and the formation of oxygen vacancies, thereby improving the redox properties. The CuCe samples exhibit higher catalytic performance compared to bare CeO_2_ and CuO catalysts. This is ascribed to the synergistic interaction between copper oxide and ceria. In particular, the Cu_0.1_Ce catalyst shows the highest catalytic performance (*T*_50_ = 59 °C), as well as excellent stability. The *in situ* DRIFTS results show that CO adsorbed on surface Cu^+^ (Cu^+^–CO species) can easily react with the active oxygen, while stronger adsorption of carbonate-like species causes catalyst deactivation during the reaction.

## Abstract 2

Surface Cu–O–Ce species can be reduced by CO and active oxygen can easily react with Cu^+^–CO to form CO_2_.

## Graphical abstract

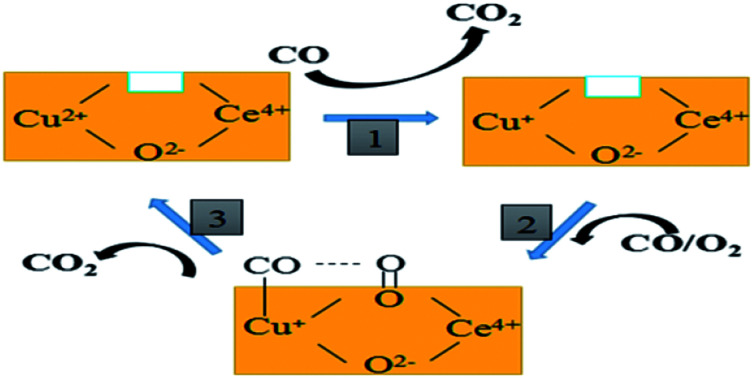

## Introduction

Carbon monoxide (CO) is a toxic gaseous pollutant produced due to the incomplete combustion of carbon-containing fuels and in other application fields.^1–3^ The majority of CO emissions come from modern automobiles; in particular, up to 80% of CO is still emitted during the cold-start stage of a vehicle, namely the first 30 s of starting a car.^4,5^ The catalytic oxidation of CO with energy-efficient and environmentally friendly features has been the most widely used control technology to remediate CO emissions.^6^ Although noble metal catalysts offer excellent catalytic properties and desirable stability, their high cost and low abundance limit their application.

Over the last few decades, ceria has been applied as an efficient three-way catalyst (TWC) for the elimination of toxic auto-exhaust gases due to its unique redox properties and high oxygen storage capacity (OCS) due to the facile redox cycle between the Ce^3+^ and Ce^4+^ in the structure.^7–9^ In particular, much attention has been paid to copper-doped ceria-based catalysts because of their unique activity for ambient CO oxidation.^10–14^ It should be noted that synergistic redox properties are produced upon the formation of CuO–CeO_2_ interfacial sites, which are considered to be the active sites for the CO oxidation reaction.^15^ Moreover, the substitution of Ce^4+^ cations with Cu^2+^ promotes the formation of structural defects and oxygen vacancies on the catalyst surface, which can favor the generation and mobility of charged species, such as electrons or oxygen anions in the solid catalyst.^16^ Thus, defect sites and oxygen vacancies are among the most desirable active species for oxidation catalysis.^17,18^ Nevertheless, some recent work has suggested that more surface oxygen vacancies may actually decrease the intrinsic activity of pure CeO_2_.^16,19^ On the other hand, the catalytic activities of nanocrystalline mesoporous materials are reported to be superior to those of their nonporous counterparts of the same material, due to their tunable porosity, surface oxygen vacancies, and large surface areas, which promote lattice oxygen mobility.^20,21^ For a gas–solid reaction, the large surface area and high mesoporosity of the catalyst favors easy access of the reactants to the active sites and diffusion of the products,^22^ thus improving the reaction efficiency on the material surface and resulting in enhanced catalytic performance. It should be noted that the preparation method could exert a basic influence on the structural properties of the catalysts, like surface area, component dispersion and strength of interaction, which in turn determine the redox properties and reactivity of the final catalysts.^23^ In order to synthesize a mesoporous material, the cationic surfactant CTAB is used as a soft template in the precipitation process, which may serve as a growth controller and an agglomeration inhibitor, by forming a covering film on the newly formed oxide crystal. In addition, the cationic surfactants could reduce the surface tension of the solution and readily be adsorbed onto the surface of the oxide through strong electrostatic interactions. Ma *et al.* have synthesized CuO/CeO_2_ bimetal oxides by using CTAB as the structure directing agent, which possesses a high surface area and disordered worm-like mesoporous structure.^24^ Wu *et al.* have reported that FeMnTiO_*x*_ mixed oxide catalysts prepared by a CTAB-assisted process^25,26^ show obvious advantages in contrast to those prepared by other methods. In our previous work, we have also successfully synthesized mesoporous ceria-based catalysts doped with different transition metals using CTAB as a soft template.^27^

Though a number of Cu-doped ceria-based catalysts have already been reported, a comparative study is necessary to determine the dependence of the mesoporous structure on the amount of dopant and the influence of the dopant on the catalytic activity, which have not been well explored yet. In addition, a detailed investigation of the mechanism and catalytic application of doped ceria-based oxides for enhanced CO oxidation activity is still needed.

In this work, a set of CuCe catalysts with different molar ratios were prepared using a facile CTAB-assisted method. The Cu/Ce atomic ratio is optimized by using CO oxidation as a model reaction, to probe the reaction of more complex oxidation processes. Systematic techniques were employed to determine the structural/textural properties of the synthesized catalysts. Moreover, the critical factors for the superior catalytic activity were thoroughly investigated in order to correlate the catalytic behavior with the nature of the catalysts.

## Experimental

### Catalyst preparation

The catalysts were synthesized using the co-precipitation method applying CTAB as a soft template.^27^ Cu(NO_3_)_2_·3H_2_O (AR) and Ce(NO_3_)_3_·6H_2_O (AR) were added to deionized water at molar ratios of Cu/(Cu + Ce) = (0, 0.05, 0.1, 0.2, 0.3, and 1). Then, the salt solution was added to the CTAB solution (with a 1 : 1 ratio of CTAB to metal ions) under vigorous stirring for 30 min, giving a clear homogeneous solution. Afterwards, a 1 M NaOH aqueous solution was added dropwise into the mixed solution under vigorous stirring until pH = 11. After stirring in the mother liquor for 3 h at 80 °C, the obtained precipitate was then filtered and repeatedly washed with sufficient deionized water and alcohol. Finally, the solid product was dried at 110 °C for 12 h in an electrical oven and then calcined at 550 °C for 6 h. The catalysts were denoted as CeO_2_, Cu_0.05_Ce, Cu_0.1_Ce, Cu_0.2_Ce, Cu_0.3_Ce, and CuO, respectively. For comparison, the reference CeO_2_ and Cu_0.1_Ce samples were synthesized by the co-precipitation method without CTAB (denoted as CeO_2_-co and Cu_0.1_Ce-co), and the mechanical mixture CuCe was prepared with a 1 : 9 ratio of CuO to CeO_2_ in order to compare it with the Cu_0.1_Ce sample (denoted as Cu_0.1_Ce-m).

### Catalyst characterization

Powder XRD patterns were acquired using a BRUKER-AXS D8 Advance X-ray Diffractometer using Cu Kα radiation (*λ* = 0.15418 nm), operated at 40 kV and 40 mA. The diffractograms were recorded in the scanning angle (2*θ*) range of 10–80° with a step size of 0.02° and a scanning speed of 6° min^−1^.

The average particle size and size distribution of the catalysts were observed by using the dynamic light scattering (DLS) technique on a Zetasizer Nano-S system (Malvern 3690 Instruments, UK). These measurements throw light on the stability and particle size distribution in fluids.

The morphology of the samples was analyzed using a transmission electron microscope (TEM, HT7700, Hitachi, Japan) operated at an acceleration voltage of 100 kV.

The textural properties of the as-prepared samples were determined from N_2_ adsorption–desorption isotherms at −196 °C on a Micrometrics ASAP 2020 analyzer. Prior to each measurement, the samples were degassed under vacuum at 300 °C for 5 h to remove the residual moisture. The specific surface areas were calculated using the Brunauer–Emmett–Teller (BET) method. The pore volumes and sizes were estimated from the pore size distribution curves determined from the adsorption isotherms using the Barrett–Joyner–Halenda (BJH) method.

The Raman spectra were recorded at room temperature (RT) on a LabRAM HR Evolution Raman spectrophotometer (Horiba Scientific, America) equipped with a TE-cooled charge coupled device (CCD) detector, using monochromatic radiation emitted by a laser (*λ* = 532 nm).

The temperature-programmed reduction with H_2_ (H_2_-TPR) experiments were performed in a fixed-bed quartz reactor with a thermal conductivity detector (TCD) to monitor the H_2_ consumption. Prior to the test, a 50 mg sample (40–80 mesh) was pretreated *in situ* at 500 °C for 30 min in a flow of He and cooled to RT. Finally, the reactor temperature was raised up to 900 °C at a heating rate of 10 °C min^−1^ in 5 vol% H_2_/N_2_ (50 mL min^−1^).

### *In situ* DRIFTS analysis

The diffuse reflectance infrared Fourier transform spectra (DRIFTS) were recorded on a Nicolet iS50 spectrometer equipped with a temperature-controllable diffuse reflection chamber and a high sensitivity MCT detector. The catalysts were pretreated in a He stream at 500 °C for 30 min to clean the surface of the catalysts and then cooled down to RT. The background spectrum was taken at each temperature of interest. The sample wafers were exposed to a controlled stream of 2000 ppm CO or/and 5 vol% O_2_/He at a flow rate of 50 mL min^−1^. All the spectra were determined by accumulating 32 scans at a resolution of 4 cm^−1^ as a function of adsorption temperature.

### Evaluation of catalytic activity

The catalytic activity of CO oxidation was evaluated in a fixed-bed reactor (i.d = 6 mm) using 100 mg of catalyst (40–80 mesh). The catalysts were pretreated in He (100 mL min^−1^) at 500 °C for 30 min to remove surface-adsorbed species. After cooling down to RT, the inlet gas was switched to 2000 ppm CO/5 vol% O_2_ in He with a flow rate of 50 mL min^−1^ and then a TPO test was carried out in the temperature range from RT to 700 °C at a heating rate of 4 °C min^−1^. The outlet gas from the reactor was analyzed online by a gas chromatograph (GC) (SP-6890, Shandong Lunan Ruihong Chemical Instrument Corporation, China). The values for the conversion of CO in the CO oxidation process are defined as follows:CO conversion (%) = (*F*^in^_CO_ − *F*^out^_CO_)/*F*^in^_CO_ × 100%,where *F* is the (inlet or outlet) molar flow of CO. Temperatures corresponding to 50% and 90% CO conversion (denoted as *T*_50_ and *T*_90_, respectively) were taken as indices of the catalytic activity.

## Results and discussion

### XRD and DLS analysis

**Fig. 1:**
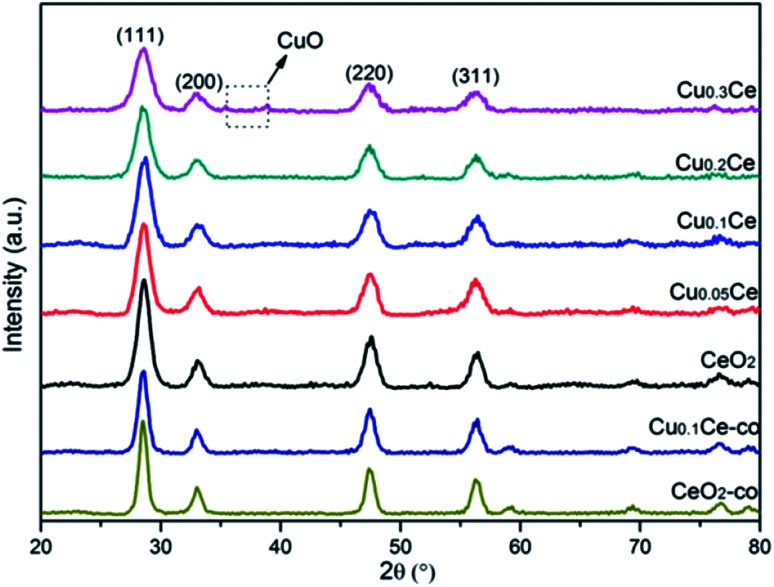
XRD patterns of CuCe catalysts with different CuO contents.

The XRD patterns of the samples with different molar ratios of Cu to Ce are shown in Fig. 1. It can be observed that all of the diffraction peaks of the samples are characteristic of the typical cubic fluorite phase of ceria (JCPDS no. 65-5923). Additionally, it can be noted that the diffraction peaks of these samples are slightly shifted to higher Bragg angles due to the incorporation of Cu ions into the ceria lattice. This interesting observation may lead to the decrease in the lattice parameter due to the smaller size of the copper ions (Table 1). Furthermore, the changes in the lattice parameter could be directly related to the concentration of oxygen vacancies and the strong interaction of CuO with the ceria phase.^28^ The changes in these distinct features, including peak shift and lattice parameter, compared to cubic CeO_2_ provide evidence of the formation of CuCe solid solutions with Cu doped into the CeO_2_ lattice, or/and the existence of some well dispersed CuO species on the surface of CeO_2_.^29–31^ However, at a higher Cu content (namely Cu_0.3_Ce), very weak reflections at 35.5° and 38.6° attributed to CuO can be detected, which indicates that when the copper doping is higher than the limit of Cu^*x*+^ incorporated into the CeO_2_ lattice, the remainder could reside on the CeO_2_ surface in the form of bulk CuO crystallites. On the other hand, the diffraction peaks of these CuCe samples are broadened resulting from the crystallite nano dimension effect compared with CeO_2_,^32^ which is supported by the calculated crystallite size of the samples based on the 111 plane using the Debye–Scherrer equation (Table 1). The smaller crystallite size of the CuCe mixed oxides may be due to the introduction of Cu^*x*+^ into the lattice of CeO_2_, which thereby suppresses the crystallite growth.^33^ For the comparison samples, the XRD peaks of the CeO_2_-co and Cu_0.1_Ce-co samples also match well the standard diffraction data for fluorite ceria; however, a larger crystallite size for these samples can be observed compared with the corresponding CeO_2_ and Cu_0.1_Ce samples, which illustrates that CTAB may serve as a growth controller of crystallites in the precipitation process.

**Table 1:** Solid properties of the catalysts

Samples	2*θ*^a^ (°)	*a* b (Å)	*X*_s_ c (nm)	*S*_BET_ d (m^2^ g^−1^)	*V*_p_ e (cm^3^ g^−1^)	*D*_p_ f (nm)
CeO_2_	28.464	5.4167	7.3	105	0.117	4.5
Cu_0.05_Ce	28.531	5.4076	6.6	113	0.177	6.3
Cu_0.1_Ce	28.564	5.4031	6.5	121	0.194	6.4
Cu_0.2_Ce	28.537	5.3990	6.2	126	0.194	6.1
Cu_0.3_Ce	28.556	5.3928	5.5	131	0.196	6.1
CeO_2_-co	28.490	5.4191	9.7	57	0.053	3.7
Cu_0.1_Ce-co	28.501	5.4078	8.4	68	0.085	4.9

a 111 crystal face.

b Lattice parameter calculated from the characteristic XRD peaks of CeO_2_.

c Mean crystallite size.

d BET specific surface area.

e Total pore volume.

f Average pore diameter.

**Fig. 2:**
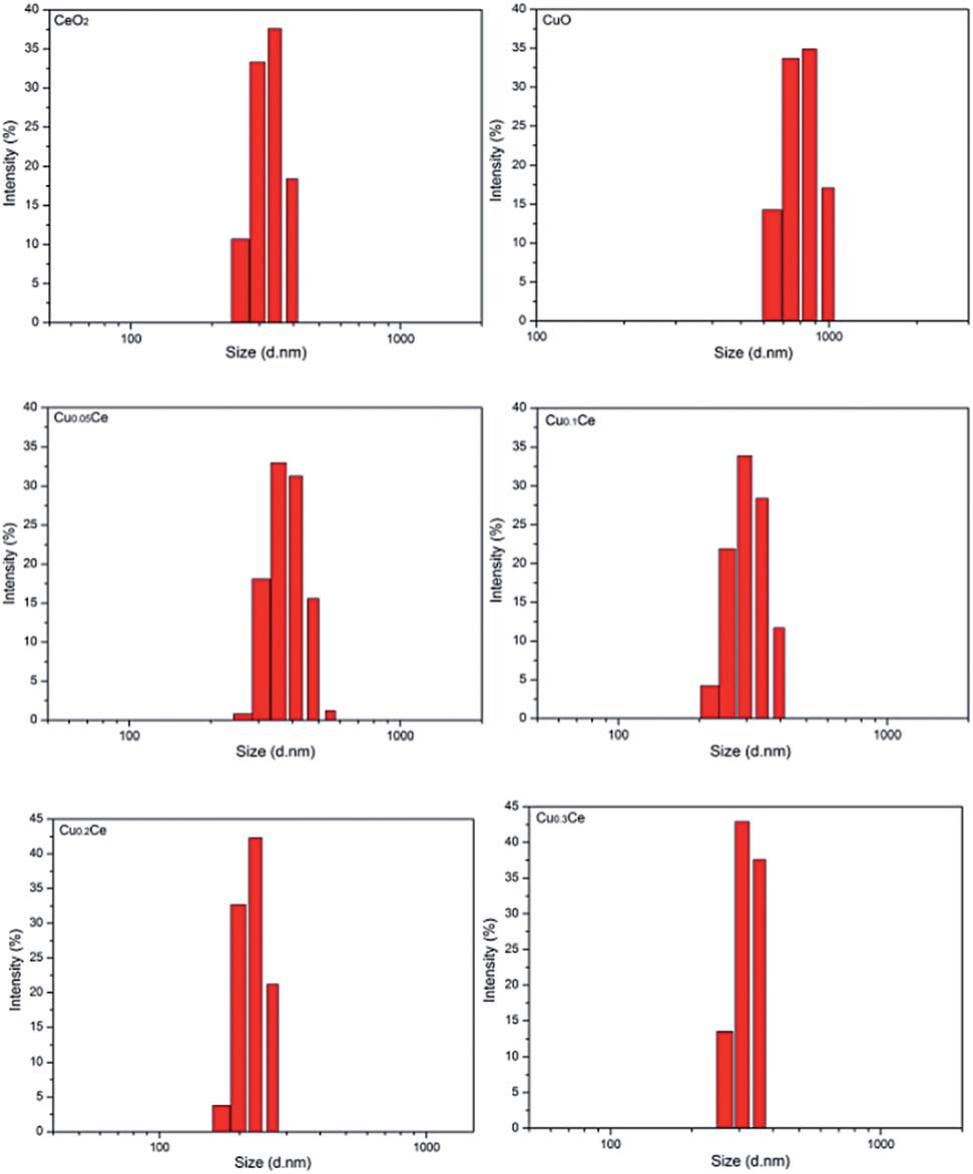
Particle size distribution of the obtained catalysts.

The particle sizes of the samples were also estimated by performing the DLS technique, which gives the particle size in the form of hydrodynamic radius, considering each particle as a separate sphere in Brownian motion. The size distribution is shown in Fig. 2 in the form of a histogram. The pure CuO sample has a larger particle size with an average value of about 811 nm (calculated based on Fig. 2). A narrow range of particle size distribution can be observed in CeO_2_, Cu_0.05_Ce, Cu_0.1_Ce, Cu_0.2_Ce and Cu_0.3_Ce, whose average particle sizes are 327 nm, 388 nm, 307 nm, 224 nm and 301 nm, respectively. These results reveal that an appropriate amount of copper doping can influence the particle growth and inhibit the agglomeration of particles due to the synergistic interaction between copper oxide and cerium oxide. Furthermore, these size values are also much larger than those calculated from the XRD results, which is because the crystallite size from XRD is the average grain size while the particle size from DLS analysis is the size of the particles composed of multiple grains.^34^

**Fig. 3:**
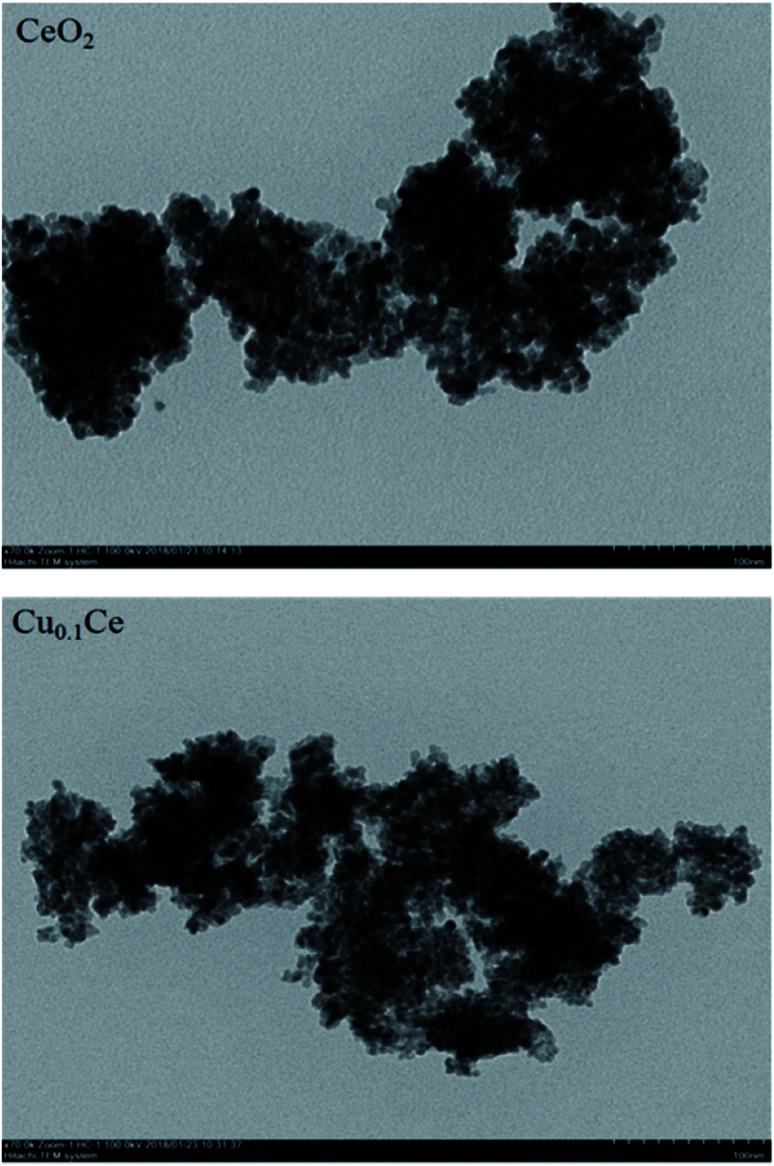
TEM images of CeO_2_ and Ce_0.1_Cu.

TEM was undertaken to further evaluate the morphological properties of the synthesized representative catalysts, as shown in Fig. 3. A disordered wormhole-like mesostructure, aggregated by metal oxide nanoparticles with a diameter of several nanometers, can be directly observed for the samples. The accessible pores are connected randomly, lacking discernible long-range order in the pore arrangement among the nanoparticles. Furthermore, the results also confirmed that the particle size from the DLS analysis is the size of the particles composed of multiple grains.

### Textural characteristics

**Fig. 4:**
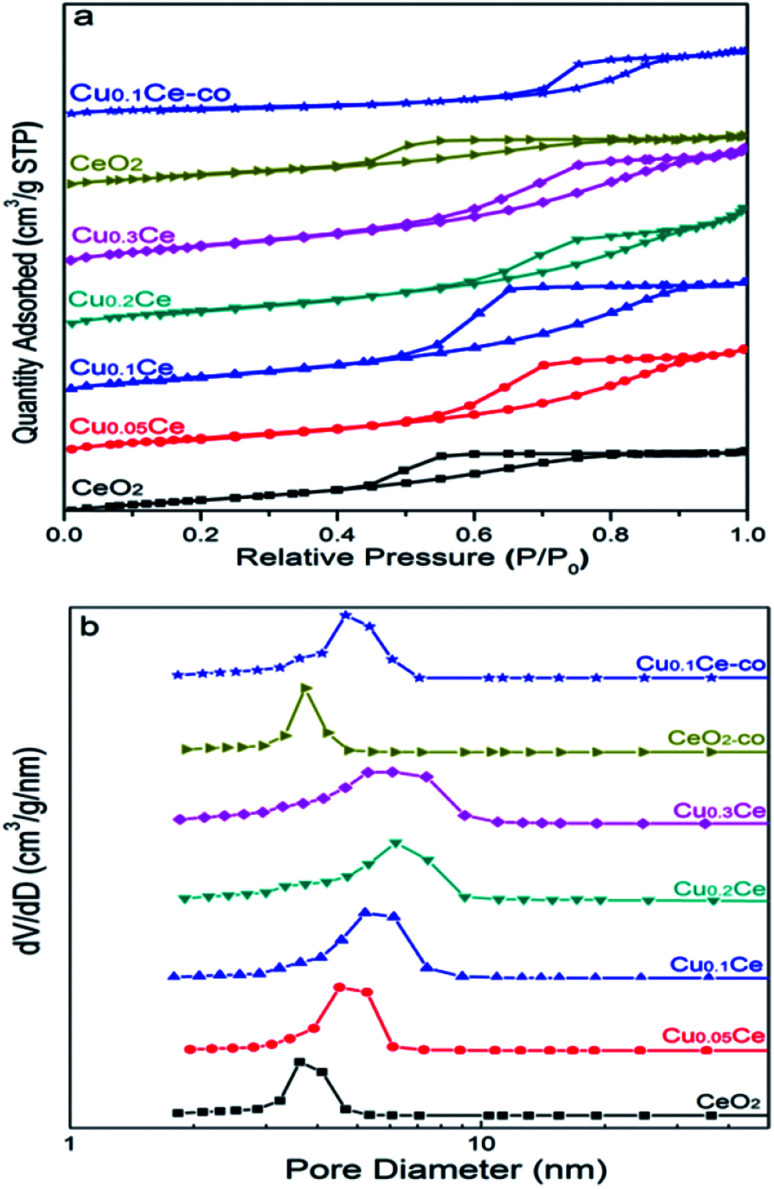
N_2_ sorption isotherms (a) and pore size distributions (b) of the CuCe catalysts with different CuO contents.

The nitrogen adsorption/desorption isotherms of the CuCe catalysts are plotted in Fig. 4(a). All the samples present classical type IV isotherms with well-defined H2-type hysteresis loops, confirming the formation of mesoporous materials according to IUPAC classification. The H2 type of hysteresis loop reflects a complex pore structure, including typical inkpot-shaped pores, tubular holes with an asymmetrical distribution of pore diameter and the mesopores of closely packed spherical particle, *etc.* As can be seen in Fig. 4(b), the mesopores in the Ce-based samples are very similar and uniform, and the most probable pore diameter of the catalyst is in the range of 4–6 nm. This result is consistent with the TEM result.

The corresponding results of textural properties are summarized in Table 1. It can be seen that the specific surface area of pure CeO_2_ is 105 m^2^ g^−1^, and the BET surface areas of the catalysts increase from 105 to 131 m^2^ g^−1^ with increasing Cu content, indicating that the introduction of Cu ions into the ceria lattice diminished the crystallite size, resulting in an increase in the surface area, which is in accordance with the XRD results.^35^ Moreover, as the Cu content increased, the pore size and pore volume of the samples remained stable at around 0.194 cm^3^ g^−1^ and 6 nm, respectively. For comparison, the surface area of CeO_2_-co and Cu_0.1_Ce-co is only 57 m^2^ g^−1^ and 69 m^2^ g^−1^, respectively, which is smaller than that of CeO_2_ (105 m^2^ g^−1^) and Cu_0.1_Ce prepared with CTAB (121 m^2^ g^−1^). Moreover, the samples prepared without CTAB also possess a smaller pore size and pore volume compared with the corresponding sample using CTAB. These results indicate that the use of CTAB in the precipitation process favors the synthesis of mesoporous materials with a large surface area. The large surface area and high mesoporosity of the catalyst allows easy access of reactants to the active sites and diffusion of products from the catalyst surface.

### Raman spectroscopy

**Fig. 5:**
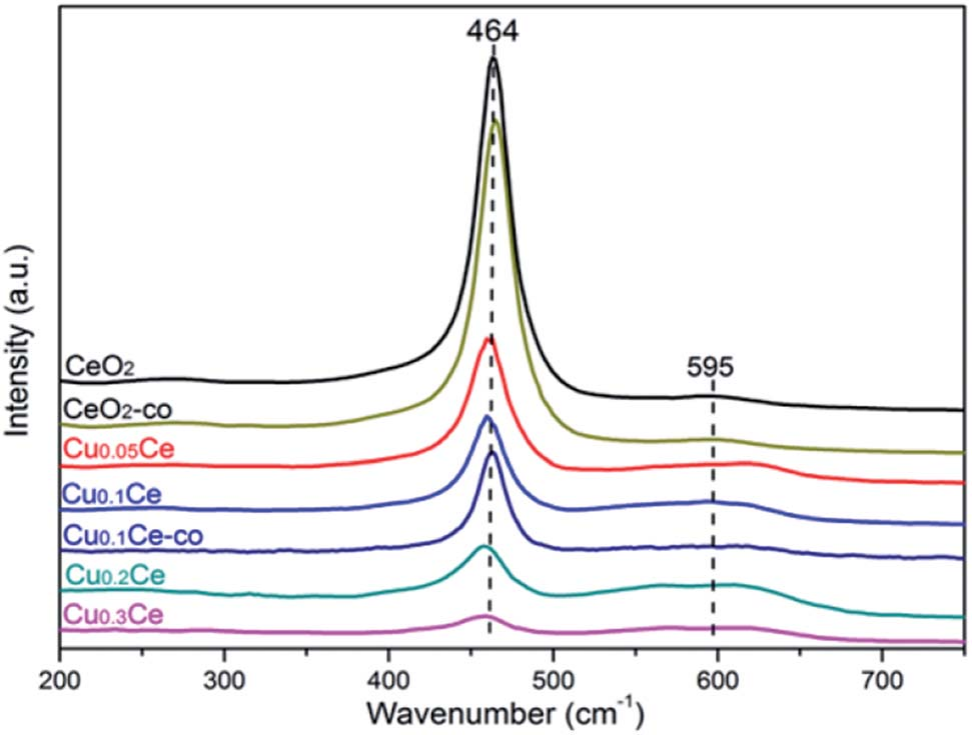
Raman spectra recorded at ambient conditions with the obtained samples.

Raman spectroscopy allows us to probe the presence of oxygen defects in ceria-based materials. It can be seen from Fig. 5 that all samples exhibit a prominent and symmetrical band at 464 cm^−1^ corresponding to the triply degenerated F_2g_ Raman active mode of fluorite-type structure CeO_2_ and a weak band at 595 cm^−1^ attributed to the presence of Frenkel-type anion defects in the ceria lattice.^16,36^ In the latter case, an oxygen ion is displaced from its lattice position to an interstitial position, thus leading to a vacancy at its initial position and a defect at the interstitial site. In addition, the ratio between the areas of the peaks at 595 cm^−1^ and 464 cm^−1^ (noted as *A*_595_/*A*_464_) is the most appropriate way to compare the concentration of oxygen vacancies in the solid solutions: the higher the value of *A*_595_/*A*_464_, the higher the amount of oxygen vacancies.^33,36^ The calculated results are summarized in Table 2. The *A*_595_/*A*_464_ values follow the order: Cu_0.3_Ce > Cu_0.2_Ce > Cu_0.1_Ce > Cu_0.05_Ce > CeO_2_, and the results suggest that the incorporation of Cu ions into the lattice of CeO_2_ favors the formation of additional oxygen vacancies. Obviously, the relative concentration of oxygen vacancies of Cu_0.1_Ce-co is lower than that of Cu_0.1_Ce. On the other hand, with increasing Cu content, the F_2g_ band of CeO_2_ becomes weaker and shifts distinctly toward lower wavenumbers, and the red shift of this band is connected with the lattice shrinkage due to the incorporation of Cu ions into the CeO_2_ lattice. These results are in agreement with the analysis results of XRD.

**Table 2:** Structure parameters and catalytic activity of the catalysts

Samples	*A*_595_/*A*_464_^a^	H_2_ consumption^b^ (mmol g^−1^)	H_2_/Cu ratio^c^	*T*_50_ d (°C)	*T*_90_ d (°C)
CeO_2_	0.11	—	—	315	411
Cu_0.05_Ce	0.15	0.76	1.216	101	154
Cu_0.1_Ce	0.31	1.58	1.264	59	92
Cu_0.2_Ce	0.66	2.41	0.964	70	101
Cu_0.3_Ce	1.07	3.60	0.960	70	101
CeO_2_-co	0.08	—	—	351	437
Cu_0.1_Ce-co	0.22	1.22	0.976	76	108

a The area ratio of the Raman bands at 595 and 464 cm^−1^.

b The total H_2_ consumption of the reduction peaks from RT to 350 °C.

c The ratio of actual amount of H_2_ consumption to theoretical amount of H_2_ consumption.

d Temperature for 50% and 90% conversion for CO oxidation.

### H_2_-TPR

**Fig. 6:**
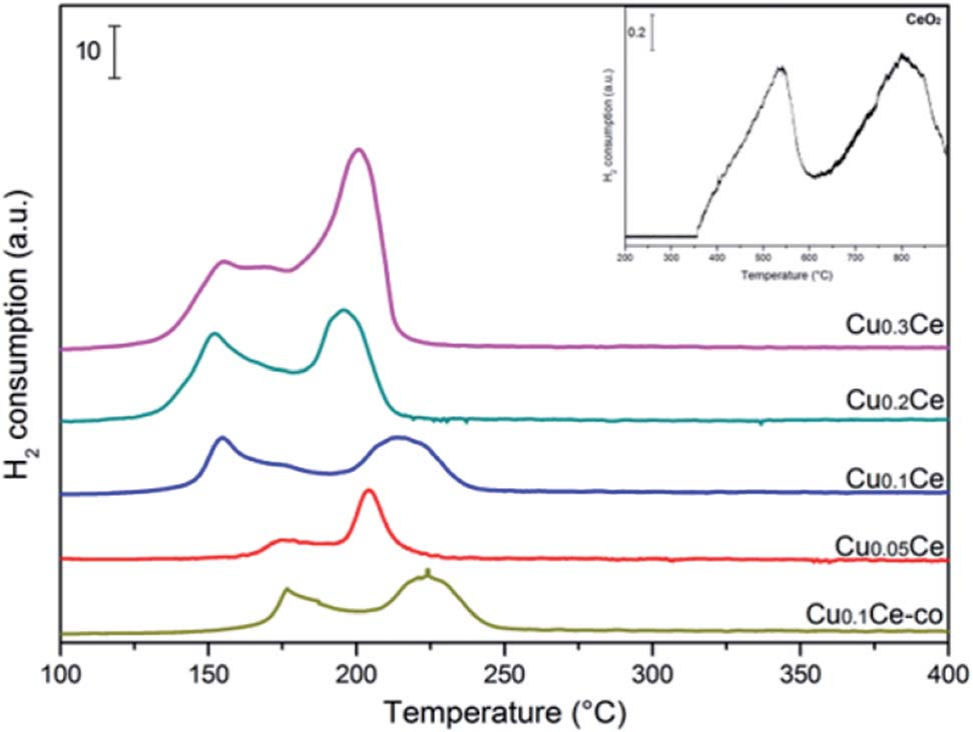
H_2_-TPR profiles of the various CuCe catalysts.

The redox properties of the catalysts were characterized by H_2_-TPR. As can be seen in Fig. 6, pure CeO_2_ exhibits two reduction peaks due to the surface and bulk reduction, which are centred at 500 °C and 800 °C, respectively. The difference in these two successive reduction temperatures is due to the different binding energy of oxygen to cerium cations in the lattice.^37^ A well-defined two-step reduction profile (designated as α and β) is observed for all CuCe catalysts in the range of 100–250 °C, which indicates that there are at least two copper species in the catalysts. It has been reported that the α and β peaks can be ascribed to the reduction of finely dispersed CuO species strongly interacting with the CeO_2_ and the reduction of larger CuO particles weakly associated with CeO_2_, respectively.^12,24^ The α and β peaks coexist in all TPR profiles even at 5 mol% CuO content, and the area of peak β is always larger than that of peak α. With increasing CuO content from 5 to 30 mol%, the intensities of the α and β peaks increase. When the CuO content increases up to 30 mol%, the intensity of the β peak continually increases and the α and β peaks coalesce and overlap to some extent, which could be due to some larger CuO particles occurring, corresponding to the fact that a separate CuO phase is obviously detected by XRD over the Cu_0.3_Ce sample. It can be clearly seen that with an increase in Cu amount from 5 to 10 mol%, peak α moves from 175 °C to 154 °C. It should be noted that the reduction peak temperature of Cu_0.1_Ce is much lower than that of Cu_0.1_Ce-co, which suggests that the redox property of Cu_0.1_Ce with more oxygen vacancies is stronger than that of Cu_0.1_Ce-co. On a further increase in the Cu amount, it stabilizes at around 154 °C. Furthermore, no reduction peak can be seen for CeO_2_ at a temperature less than 400 °C. As is well known, the reduction profile of pure CuO is characterized by a single peak at about 300 °C. The H_2_-TPR peak temperature of the CuCe catalysts is much lower than that of pure CuO and CeO_2_. This finding suggests that a strong synergistic interaction between ceria and copper oxide takes place, namely, the Ce–O bond is weakened by the substitution of ceria and Cu–O–Ce structures are formed on the surface, leading to an easier reduction of both CeO_2_ and CuO.

Quantitative analysis of the H_2_ consumption from RT to 350 °C was carried out (Table 2) and the results show that the H_2_-consumption progressively increases with the Cu-content, thus confirming the beneficial role of copper towards the reducibility of ceria-based catalysts. On the other hand, for Cu_0.05_Ce and Cu_0.1_Ce samples, the ratio of actual amount of H_2_ consumption to the theoretical amount of H_2_ consumption is larger than 1, suggesting that the reduction peak areas come from the reduction of CuO, but also CeO_2_ takes part in the reaction in the range of lower temperature due to the strong synergistic interaction. However, the H_2_/Cu ratio is less than 1 for the Cu_0.2_Ce and Cu_0.3_Ce, which implies that more large CuO particles weakly interact with CeO_2_, confirming that the reduction of surface oxygen species could be hindered due to the formation of excess CuO crystallites with higher Cu-content, which reduces the interaction of Cu–O–Ce structures. In general, the high value of H_2_ consumption implies that the samples contain more reducible oxygen species. As discussed in the XRD section, the oxygen vacancies would be generated once the Cu ions are incorporated into the CeO_2_ lattice, which is also confirmed by Raman analyses. The oxygen vacancies would result in charge imbalance and lattice distortion of CeO_2_, thereby creating more active sites on the surface that adsorb more oxygen molecules. The adsorbed oxygen molecules are very reactive oxygen species and are readily reduced at relatively low temperatures.

### Catalytic performance

**Fig. 7:**
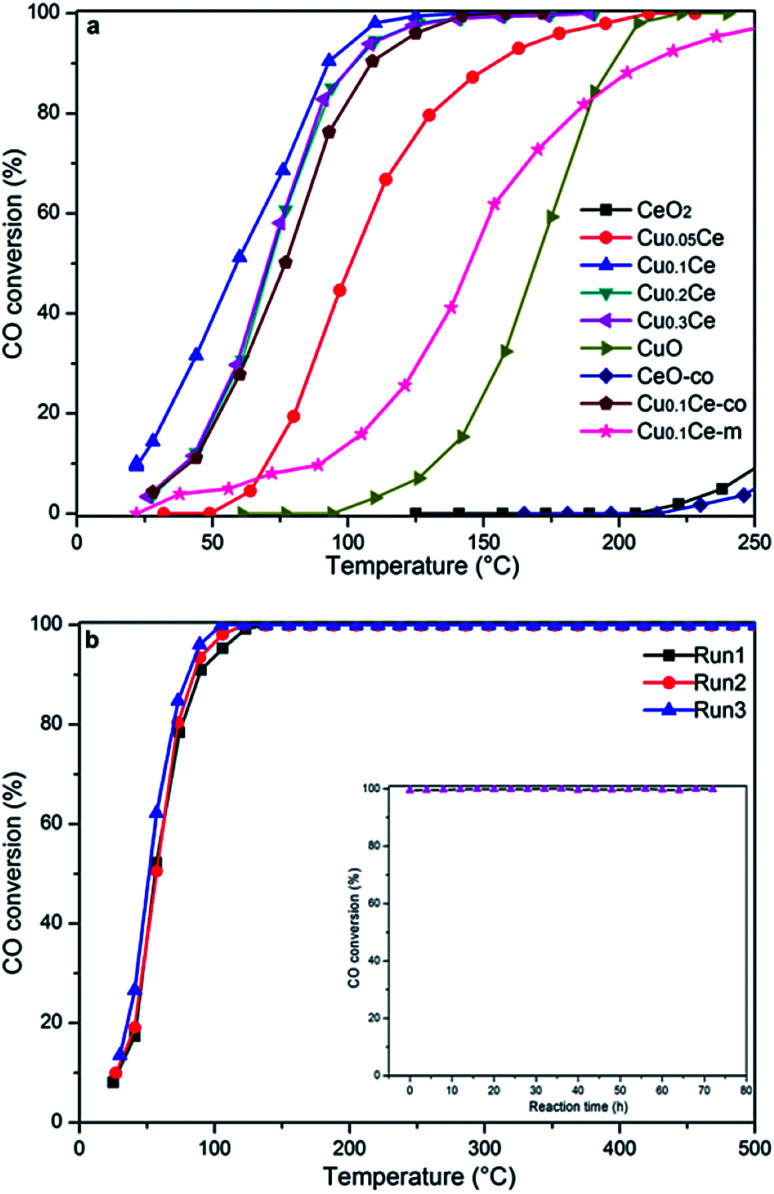
The catalytic activities of the CuCe catalysts for CO oxidation (a) and stability study of the Cu_0.1_Ce catalyst for three successive catalytic cycles (b). Inset: effect of time-on stream on the catalytic activity.

The activity results corresponding to various samples are represented in Fig. 7(a). For comparison, CO oxidation was also performed with CeO_2_-co, Cu_0.1_Ce-co and Cu_0.1_Ce-m samples. It is obvious that pure CeO_2_ exhibits almost no activity in this temperature window and CuO shows modest catalytic activity. The CeO_2_-co sample exhibits lower catalytic performance compared with the CeO_2_ prepared with CTAB as expected. The increased catalytic performance of the CeO_2_ sample can be ascribed to the mesostructures and high surface area. In contrast, when the content of Cu reaches 5 mol%, its catalytic activity is improved to a certain extent, suggesting that appropriate doping of CuO into CeO_2_ contributes to an increase in catalytic performance for CO. The CO conversions gradually increase with an increase in the content of Cu until 10 mol% (Cu_0.1_Ce). The mechanical mixture Cu_0.1_Ce-m also exhibits enhanced catalytic performance compared with single CeO_2_ and CuO, which confirms the synergistic interaction between CuO and CeO_2_. For comparison purposes, we have calculated the temperature at which 50% conversion occurs, denoted as *T*_50_. The Cu_0.1_Ce catalyst exhibits the best catalytic activity and the *T*_50_ is 59 °C, which is also superior to that of the Cu_0.1_Ce-co sample. With further increasing CuO content, the catalytic performance of the samples decreases slightly. The catalytic activity of CO oxidation in terms of *T*_50_ is in the order: Cu_0.1_Ce > Cu_0.2_Ce = Cu_0.3_Ce > Cu_0.05_Ce > CuO > CeO_2_.

The catalytic oxidation of CO on an active metal supported ceria catalyst is proposed to involve reductive and oxidative steps, which was interpreted by the classical Mars-van Krevelen and Langmuir–Hinshelwood mechanisms. In the former mechanism, the adsorbed CO (CO_ad_) is oxidized by lattice oxygen (O_L_). In the latter case, the CO_ad_ is oxidized mainly by adsorbed oxygen (O_ad_), which is the rate-determining process. Therefore, the mobility and/or reducibility of the active oxygen (O_ad_ and/or O_L_) is of crucial importance for the CO oxidation reaction. Following the previous characterizations, some reasonable analyses under the current conditions are concluded to further illustrate factors that affect the catalytic activity. The results of XRD suggest that the incorporation of Cu ions with smaller ionic radii into the ceria lattice results in defective solid solutions. The synergistic interaction between CuO and CeO_2_ has a positive effect on the generation of surface oxygen vacancies, which has also been confirmed by the Raman spectroscopy study. Gaseous oxygen in the feed gases could be absorbed at surface oxygen vacancies and sequentially activated as active oxygen (O_2_^−^) by activation of the catalysts. In addition, the generated oxygen vacancies acting as active sites could also improve the mobility of oxygen species from the bulk to the surface, which is well shown by the results of the H_2_-TPR experiment. The large surface area and high mesoporosity of the doped catalyst allows easy access of reactants (CO) to the active sites and diffusion of products (CO_2_) from the catalyst surface. Thus, the Cu-doped ceria-based catalysts exhibit better catalytic performance than pure oxides.

According to the results of structure and Raman characterization, the BET surface areas and oxygen vacancies of the doped catalysts increase with the Cu content, but the catalytic activity of Cu_0.2_Ce and Cu_0.3_Ce is slightly decreased compared with Cu_0.1_Ce. In this sense, the surface area is not the main factor influencing the catalytic activity. Furthermore, more surface oxygen vacancies may even decrease the intrinsic activity of the catalysts.^16^ These results indicate that the role of ceria surface oxygen vacancies in CO oxidation may be more complex than ever expected. Given that the H_2_/Cu ratio of Cu_0.1_Ce (1.264) is larger than 1, the interface between CuO and CeO_2_ has a stronger synergistic interaction for Cu_0.1_Ce, and the interaction decreases with increasing Cu content. It should be noted that the active sites of the Cu-doped ceria for CO oxidation are located in the interface between CuO and the CeO_2_ substrate.^38^ When the doping amount of CuO increases, some bulk CuO clusters could appear and cover the surface active sites,^39^ because a separate CuO phase is obviously detected by XRD over the Cu_0.3_Ce sample.

For practical application purposes, catalytic stability is an important feature for materials used in industrial applications. Therefore, CO oxidation cycling tests have been performed over the Cu_0.1_Ce catalyst as a function of temperature. An additional long-term stability test in normal feed gas was conducted at constant temperature. As shown in Fig. 7(b), the Cu_0.1_Ce catalyst exhibited high stability, and no deactivation occurred for a time-on-stream of 72 h at 120 °C (inset to Fig. 7(b)).

### *In situ* DRIFTS analysis

**Fig. 8:**
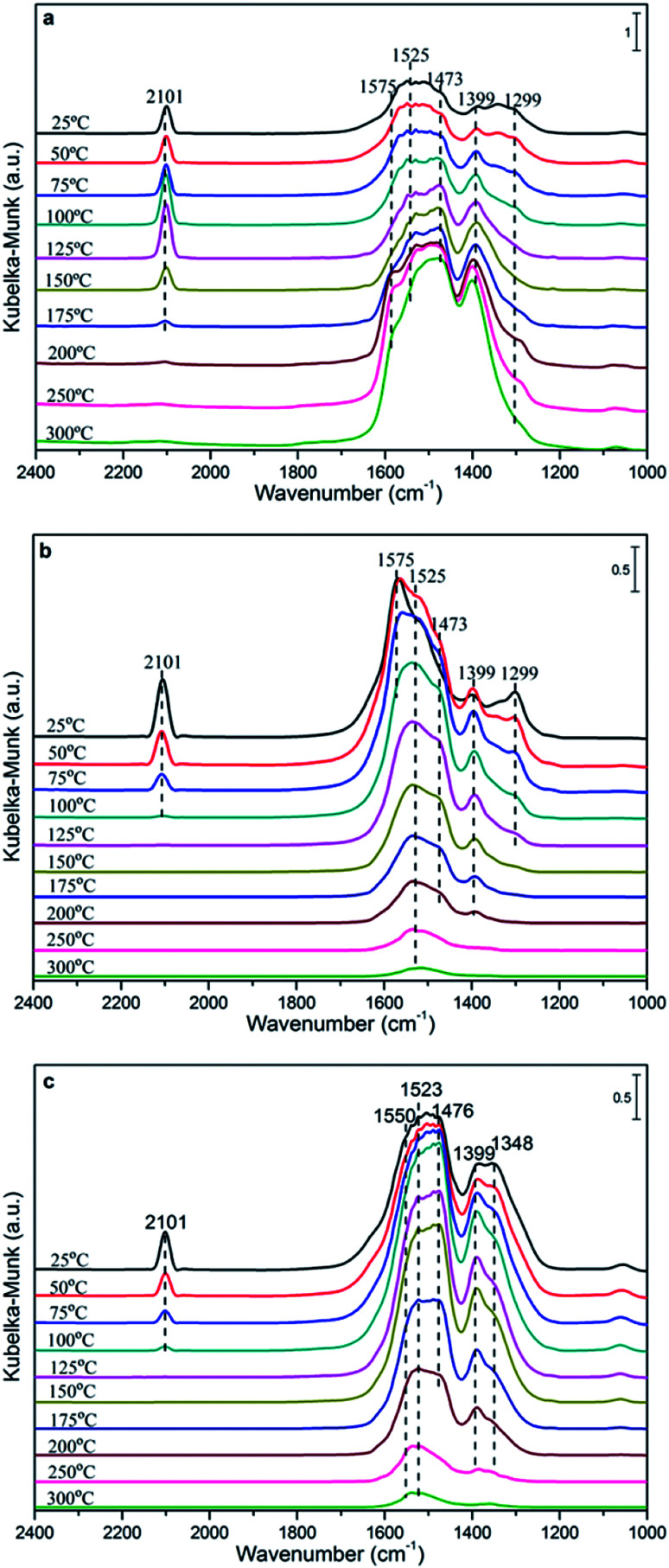
DRIFTS spectra recorded after exposure to CO on the Cu_0.1_Ce sample (a) and CO + O_2_ on the Cu_0.1_Ce (b) and Cu_0.2_Ce (c) samples at different temperatures.

In order to investigate the influencing factors of the CO oxidation activity and explore the reaction mechanism further, the *in situ* DRIFTS technique was carried out over the CuCe catalysts and the results are shown in Fig. 8.

Fig. 8(a) shows the spectra of CO adsorption over Cu_0.1_Ce with the purpose of probing the surface species. CO can absorb on either Cu surfaces to form adsorbed CO or on CeO_2_ surfaces to form various carbonate, bicarbonate and formate species in the 1700–1000 cm^−1^ range.^13,16,32,40^ It is widely reported that the bands at 1575, 1525 and 1299 cm^−1^ are ascribed to bidentate carbonate species. The bands attributed to bridged carbonate and monodentate carbonates appear at 1399 and 1473 cm^−1^. Furthermore, the intensity of the bands attributed to these carbonates enhances on further raising the temperature. In addition, bridged formate (1356 cm^−1^) is observed at low temperature and disappears when the temperature is increased up to 100 °C. In principle, the CO molecule is an electron-donor probe during adsorption, so the active interface oxygen species may capture the CO molecule, forming interface carbonate species. The peak at about 2101 cm^−1^ in the spectra should be attributed to the vibration of Cu^+^–CO, which indicates the presence of some Cu^+^ species in the sample. Generally, at ambient temperature, the adsorption of Cu^+^–CO is the most stable.^41,42^ Meanwhile, the intensity of the band for Cu^+^–CO shows a maximum at 125 °C and a disappearance at 200 °C, which may be due to the interaction of CO with Cu^2+^ and Cu^+^ ions with unsaturated coordination leading to their reduction, *i.e.*, from Cu^2+^ to Cu^+^ to Cu^0^.

The DRIFTS spectra of CO and O_2_ co-adsorption over Cu_0.1_Ce are shown in Fig. 8(b). It can be seen that the intensity of Cu^+^–CO at ambient temperature is slightly weaker than that of only CO adsorption, which could be due to the preferential adsorption of O_2_ molecules on the surface of the catalyst to form O_2_^−^ species in the oxygen vacancies, restraining the adsorption of CO.^42^ The Cu^+^–CO species weakens gradually with the increase in temperature and disappears completely at 100 °C, which suggests that gaseous CO may be completely oxidized to CO_2_ by excess O_2_. This is confirmed by the catalytic activity of CO oxidation. The bands that appeared between 1200 and 1700 cm^−1^ increase gradually and reach the maximum at 50 °C, then weaken gradually when the temperature exceeds 100 °C. This may be due to the carbonate-like species first being produced through the conversion of CO_2_ or other processes and then desorbing from the catalyst surface at higher temperatures. On the other hand, the CO conversion tend to grow slowly when the reaction temperature exceed 100 °C. It is reasonable that the carbonate species would limit the reaction rate and lead to low activity due to the produced carbonate-like species blocking some of the active sites, resulting in surface like-poisoning.^13,43^

For comparison purposes, CO and O_2_ co-adsorption *in situ* DRIFTS spectra of the Cu_0.2_Ce sample were recorded so as to explore the mechanism and better understand the difference in catalytic activity for the CO oxidation reaction. It can be seen from Fig. 8(c) that the change of the adsorbed species with temperature is similar to that of Cu_0.1_Ce. It should be noted that the band of Cu^+^–CO shows a lower intensity at RT and obviously exists at 100 °C compared to Cu_0.1_Ce. The respective intensities of these Cu^+^-carbonyls provide a measure to evaluate the potential of CO oxidation for each catalyst.^44^ That is to say, the intensity of the Cu^+^-carbonyl band may be proportional to the CO oxidation activity. The bands attributed to these carbonates and carboxylates with higher intensity reach the maximum at 100 °C, and decrease gradually with increasing temperature after 125 °C for Cu_0.2_Ce. Two new bands ascribed to bridged formate and carboxylates are observed at 1348 and 1550 cm^−1^. These observations indicate that an increase in Cu doping may improve the adsorption capacity for carbonaceous species, which could result from its larger surface area and more oxygen vacancies. In turn, the carbon deposition phenomenon could easily cover the catalyst surface, blocking some active sites and further causing catalyst deactivation during the reaction.

**Fig. 9:**
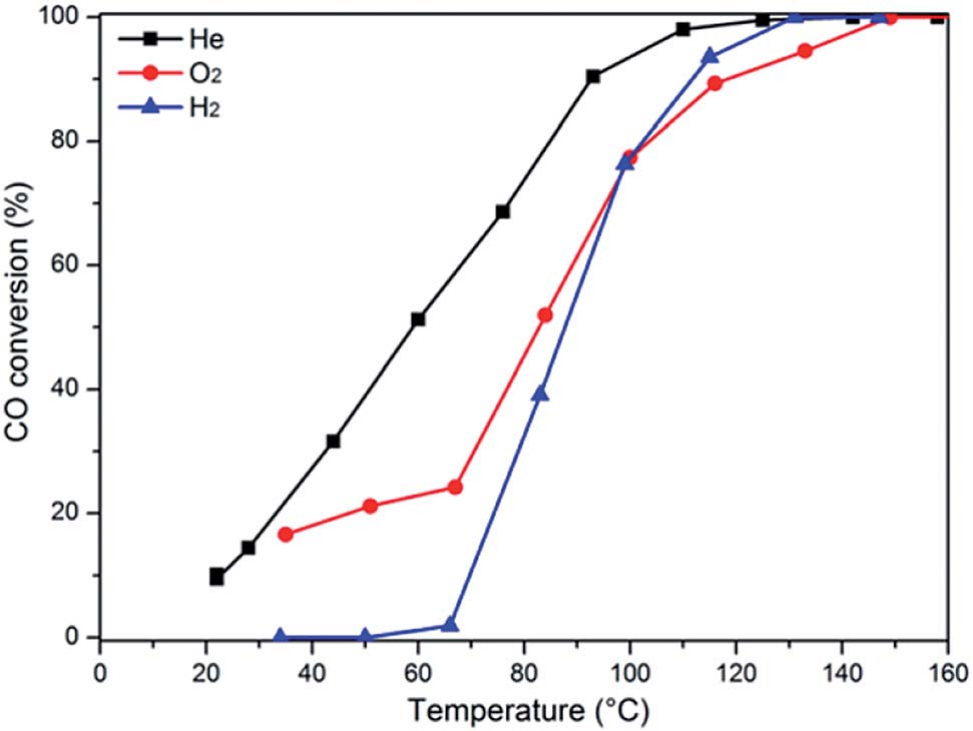
Influence of pretreatment conditions on the CO conversion.

In order to further determine the influence of the pretreatment conditions on the catalytic activity, the representative sample Cu_0.1_Ce was pretreated in different atmospheres (He, O_2_ and H_2_) before the CO oxidation reaction, as shown in Fig. 9. The observed *T*_50_ temperatures of the catalysts pretreated with He, O_2_, and H_2_ are 59 °C, 82 °C and 87 °C, respectively. Taking into account the H_2_-TPR results, in an oxidizing or reducing atmosphere, Cu^*x*+^ species could be oxidized to Cu^2+^ or reduced to Cu^0^. Thus, it can be concluded that the presence of more Cu^+^species could result in higher activity, which confirmed the DRIFTS results. Furthermore, this consequence is in accordance with the literature,^45^ which reports partially reduced copper (Cu^+^) as the most active species.

**Fig. 10:**
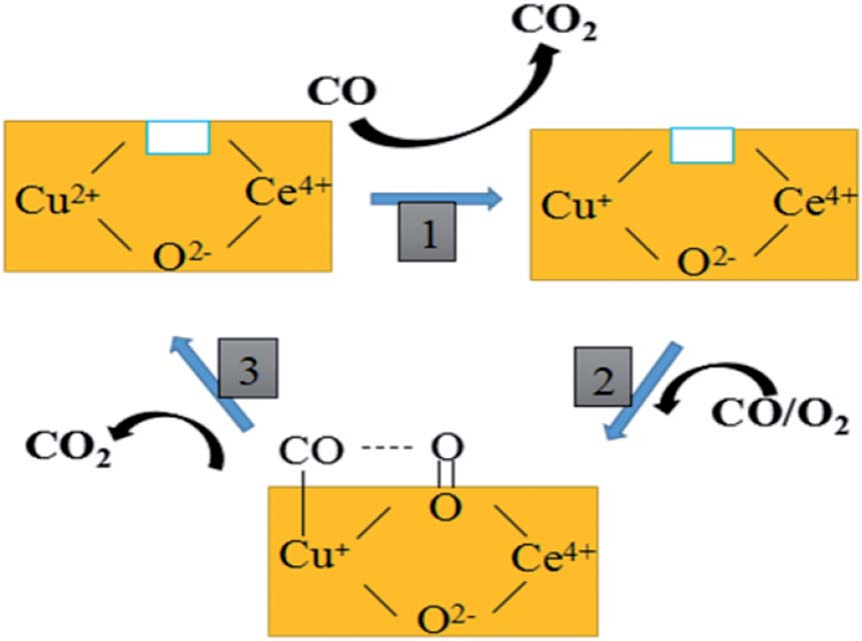
The proposed reaction mechanism of CO oxidation over the CuCe catalysts.

Based on the above discussion and previous reports, the proposed cyclic schematic representation of CO oxidation involves a combination of steps as shown in Fig. 10. In the first step, the surface Cu–O–Ce species could be reduced by CO in the mixed atmosphere to form more Cu^+^ species and surface synergetic oxygen vacancies. This process of electron transfer activates the lattice oxygen nearby the copper species. In the second step, the exposed Cu^+^ and oxygen vacancy could provide an adsorption site for CO and an activation site for O_2_, respectively. Finally, this kind of active oxygen (O_2_^−^) can easily react with CO molecules adsorbed on Cu^+^ (Cu^+^–CO) to form CO_2_*via* intermediate carbonate-like species. In short, both the lattice oxygen derived from ceria and the active oxygen (O_2_^−^) generated on the CuCe interface can react with the adsorbing CO to form CO_2_. The consumed surface oxygen can be refilled from bulk diffusion in the catalyst lattice and gaseous oxygen by the oxygen vacancies near to the copper species.

## Conclusions

A series of mesostructured CuCe catalysts were successfully prepared *via* a CTAB-assisted co-precipitation method and tested for low-temperature CO oxidation. In these catalysts, Cu could enter the CeO_2_ matrix with a fluorite-like structure to form a solid solution, but a higher Cu content causes the formation of bulk CuO. A small crystallite size (5–7 nm) and high surface area (105–131 m^2^ g^−1^) of the nanocatalysts can be obtained. The synergistic interaction of Cu and Ce species can produce large amounts of oxygen vacancies and improve the redox ability, thereby favoring the catalytic activity. Among the obtained catalysts, Cu_0.1_Ce exhibits the highest catalytic activity for CO oxidation with the lowest *T*_50_ = 59 °C and high catalytic stability. Active oxygen species from oxygen vacancies can easily react with Cu^+^–CO to produce CO_2_*via* intermediate carbonate-like species. Furthermore, the stronger adsorption of carbonate-like species causes catalyst deactivation during the CO oxidation reaction due to the fact that the carbon deposition may block some active sites.

## Conflicts of interest

There are no conflicts to declare.

## Supplementary Material
